# Subclinical Cardiac Dysfunction and Cognitive Function: A Systematic Review

**DOI:** 10.1093/geroni/igab046.2615

**Published:** 2021-12-17

**Authors:** Mario Maalouf, Raymond Farah, Sola Bahous, Kamal Matli, Adina Zeki Al Hazzouri, Christy Costanian

**Affiliations:** 1 Lebanese American University, Lebanese American University, Beyrouth, Lebanon; 2 Lebanese University, Beirut, Beyrouth, Lebanon; 3 Lebanese American University, Beirut, Beyrouth, Lebanon; 4 Columbia University, New York, New York, United States

## Abstract

Background: Cardiovascular disease, and more recently, subclinical cardiac dysfunction have both been implicated as important risk factors for cognitive decline. Several measures have been used to detect subclinical cardiac dysfunction, with global longitudinal strain (GLS) emerging as an important and more sensitive indicator than traditional measures. Yet, the association of GLS with cognitive function remains relatively unexplored. Objective: The aim of this review is to systematically summarize the literature exploring the association between GLS and cognitive function. Methods: We conducted a systematic review of the literature following PRISMA guidelines using the following databases: PubMed, OVID Medline, Embase, Web of Science, and CINAHL. Inclusion criteria were observational studies published in English, measuring GLS and assessing cognitive function through neuropsychiatric tests or brain imaging. Quality assessment was done using the Newcastle Ottawa Scale. Results: The initial search revealed 394 studies, of which three met inclusion criteria and were included for final review. The three studies included were cross-sectional and of high quality. They all reported that lower GLS scores were associated? with worse cognitive function and more brain abnormalities in both bivariate and multivariable analysis. Conclusion: Subclinical cardiac dysfunction, identified by GLS, was associated with worse cognitive function and presence of cerebral abnormality on brain imaging. The underlying mechanism could be attributed to dysfunctional autoregulatory and microvascular processes occurring in the brain vasculature. Further longitudinal studies are needed to better delineate the relationship between GLS and cognitive function.

